# TLR2^−/−^ Mice Display Decreased Severity of Giardiasis *via* Enhanced Proinflammatory Cytokines Production Dependent on AKT Signal Pathway

**DOI:** 10.3389/fimmu.2017.01186

**Published:** 2017-09-20

**Authors:** Xin Li, Xichen Zhang, Pengtao Gong, Feifei Xia, Ling Li, Zhengtao Yang, Jianhua Li

**Affiliations:** ^1^Key Laboratory of Zoonosis, Ministry of Education, College of Veterinary Medicine, Jilin University, Changchun, China

**Keywords:** *Giardia*, giardiasis, TLR2, TLR2^−/−^, AKT, cytokines

## Abstract

*Giardia* infection is one of the most common causes of waterborne diarrheal disease in a wide array of mammalian hosts, including humans globally. Although numerous studies have indicated that adaptive immune responses are important for *Giardia* defense, however, whether the host innate immune system such as TLRs recognizes *Giardia* remains poorly understood. TLR2 plays a crucial role in pathogen recognition, innate immunity activation, and the eventual pathogen elimination. In this study, we investigated the role of TLR2 as a non-protective inflammatory response on controlling the severity of giardiasis. RT-PCR analysis suggested that TLR2 expression was increased *in vitro*. We demonstrated that *Giardia lamblia*-induced cytokines expression by the activation of p38 and ERK pathways *via* TLR2. Interestingly, the expression of IL-12 p40, TNF-α, and IL-6, but not IFN-γ, was enhanced in TLR2-blocked and TLR2^−/−^ mouse macrophages exposed to *G. lamblia* trophozoites compared with wild-type (WT) mouse macrophages. Further analysis demonstrated that *G. lamblia* trophozoites reduced cytokines secretion by activating AKT pathway in WT mouse macrophages. Immunohistochemical staining in *G. lamblia* cysts infected TLR2^−/−^ and WT mice showed that TLR2 was highly expressed in duodenum in infected WT mice. Also, infected TLR2^−/−^ and AKT-blocked mice showed an increased production of IL-12 p40 and IFN-γ compared with infected WT mice at the early stage during infection. Interestingly, infected TLR2^−/−^ and AKT-blocked mice displayed a decreased parasite burden, an increased weight gain rate, and short parasite persistence. Histological morphometry showed shortened villus length, hyperplastic crypt and decreased ratio of villus height/crypt depth in infected WT mice compared with in infected TLR2^−/−^ and AKT-blocked mice. Together, our results suggested that TLR2 deficiency leads to alleviation of giardiasis and reduction of parasite burden through the promotion of proinflammatory cytokines production. For the first time, our results demonstrated that TLR2 played a negative role in host defense against *Giardia*.

## Introduction

*Giardia lamblia* is a protozoan parasite, which colonizes exclusively in the lumen of the upper small intestine. The parasite only adheres to the surface of the enterocyte but does not traverse the enterocyte barrier. It reproduces asexually and usually causes waterborne diarrheal disease in a wide array of mammalian hosts, including humans (1–3). Approximately 20–80% of humans with positive feces specimens show symptoms (epigastric pains, nausea, vomiting, and diarrhea) (4–7). Giardiasis results in weight loss, malabsorption, and failure of children to thrive (4). Since *G. lamblia*-related diseases pose a huge burden in developing countries, which humper the socioeconomic improvements and are closely associated with poverty, giardiasis has been included in the Neglected Diseases Initiative by the World Health Organization since 2006 (1, 8). So far, the immune mechanism of host resistance to the parasitic infection is still unclear.

The innate immunity is an important arm of the immune system that provides immediate non-specific defense against infections. TLRs are recognition molecules for multiple pathogens, including parasites, virus, bacteria, and fungi, which has emerged as an important step in triggering efficient inflammatory responses (9, 10). TLRs activation could lead not only to the induction of inflammatory responses but also to the development of antigen specific adaptive immunity. The stimulation of adaptive immunity initiates a range of host defense mechanisms, such as the activation of NF-κB and production of pro-inflammatory cytokines that contribute to the effective elimination of pathogenic microorganisms (11).

TLR2, one of the toll-like receptors, is expressed on the surface of certain cells, which recognizes certain pathogen-associated molecular patterns and transduces appropriate signals to immune cells, such as macrophages. TLR2 has been reported to activate MAPK, AKT, and NF-κB signal pathways (12–15), which lead to the production of pro-inflammatory cytokines (16). TLR2 can be activated by glycosylphosphatidylinositols (GPIs) presented on some protozoa and participates in the host defense against parasite infection (10), such as *Toxoplasma gondii* and *Trypanosoma cruzi* (17, 18). However, TLR2 has also been suggested to diminish the development of adaptive immune responses during experimental deep *dermatophytosis* and to act to intensify a non-protective inflammatory response (19). For *Giardia*, it has been shown that *Giardia* trophozoites lysate may interfere with the activation of dendritic cell (DC) through TLR2 *in vitro* (20). TLR2 ligand-stimulated DCs incubated in the presence of *Giardia* trophozoites lysate produced less IL-12/23 P40, IL-12 P70, and IL-23, but more IL-10 than cells incubated without the parasite (21). However, the role of host TLR2 as an inflammatory response on controlling the severity of giardiasis remains poorly understood.

In this study, the expression of TLR2, the phosphorylation of p38, ERK, and AKT, the secretion of IL-12 p40, TNF-α, IFN-γ, and IL-6 were examined in TLR2^−/−^ and wild-type (WT) mouse peritoneal macrophages stimulated with *G. lamblia* trophozoites *in vitro*, respectively. The expression of TLR2, the secretion of IL-12 p40, TNF-α, IFN-γ, and IL-6, the percentage of CD4^+^ and CD8^+^ T cell population, parasite burden, weight gain rate, parasite persistence, and histological morphometry (villus length, hyperplastic crypt, and ratio of villus height/crypt depth) were observed and compared between *G. lamblia* cysts infected TLR2^−/−^, AKT-blocked WT mice *in vivo*. The role of host TLR2–AKT signal pathway on controlling giardiasis was elucidated.

## Materials and Methods

### Ethics Statement

Wild-type female C57BL/6J mice weighing 10–12 g (purchased from Changsheng Experimental Animal Centre, Anshan, China) and TLR2^−/−^ mice weighing 10–12 g (Model Animal Research Center of Nanjing University, Nanjing, China) housed in filter-top cages in an air-conditioned animal facility in the National Experimental Teaching Demonstration Centre of Jilin University (Changchun, China). Water and normal mouse chow were provided *ad libitum*. All animal experimental procedures were performed in strict accordance with the Regulations for the Administration of Affairs Concerning Experimental Animals approved through the State Council of People’s Republic of China (1988.11.1) and with approval of the Animal Welfare and Research Ethics Committee at Jilin University (IACUC Permit Number: 20160612).

### *G. lamblia* Trophozoites Cultivation and Mice Infection

Trophozoites of *G. lamblia* WB strain (ATCC30957; American Type Culture Collection, Manassas, VA, USA) were grown for 48 h in TYI-S-33 medium (22). All mice were given antibiotics *ad libitum* in drinking water before infection: ampicillin (1 mg/ml; Sigma-Aldrich), neomycin oral solution (1.4 mg/ml; Sangon Biotech, Shanghai, China), vancomycin (1 mg/ml; Sangon Biotech, Shanghai, China), and neomycin oral solution (1.4 mg/ml; Sangon Biotech, Shanghai, China). *G. lamblia* cysts were prepared using method as described previously (23). TLR2^−/−^ and WT mice were gavaged with 5 × 10^5^ *G. lamblia* cysts in 100 µl sterile PBS (pH 7.4). An AKT inhibitor, MK-2206 (Selleck, USA), was used to blocked AKT pathway *in vivo*. At day 3 after *Giardia* infection, MK-2206 (120 mg/kg, every 2 days by p.o.) was administered to the infected WT mice (24, 25). All mice were weighed before infection and every day post infection (dpi) until sacrifice. To measure parasite loads at different times post infection, mice were euthanized. The first 3 cm of the small intestines (pylorus to ligament of Treitz) were removed, and the next 2-cm section of the duodenum was collected. The duodenum samples were opened longitudinally and cut up in 2 ml ice-cold sterile PBS (pH 7.4), and incubated on ice for 10 min. The numbers of trophozoites were counted using a hemocytometer (26).

### Isolation of Mouse Peritoneal Macrophages

Mice were euthanized, the peritoneal cavities were flushed twice with 10 ml phosphate-buffered saline (PBS, pH 7.4), and cells were collected by centrifugation at 1,000 *g* for 10 min. Then, the cell pellets were washed twice with 10 ml PBS. 2 × 10^6^ cells were plated in a well of six-well tissue culture plates (JET BIOFIL, China) in 1 ml RPMI 1640 containing 10% FBS, 2 mM l-glutamine, 100 U/ml penicillin, and 100 mg/ml streptomycin and incubated overnight at 37°C with 5% CO_2_. Cells were washed twice with sterile PBS to remove the non-adherent cells (27).

### Small-Interfering RNA Transfection

Synthetic small-interfering RNA (siRNA) specific for AKT and siCONTROL-non-specific siRNA were purchased from Ruibo Biology Company (Guangzhou, China). Mouse macrophages were plated at equal densities in six-well plates (2 × 10^6^ cells/well). Cells were transfected with siRNA-control or siRNA AKT for 24 h using Lipofectamine 2000 transfection reagent (Invitrogen) following the manufacturer’s protocol. Following transfection, cells were furthers stimulated with 1 × 10^6^ *G. lamblia* trophozoites for 18 h. The supernatants and cells were collected for ELISA and Western blot, respectively.

### Cytokines Detection by ELISA

Mouse peritoneal macrophages were seeded in six-well culture plates at a density of 2 × 10^6^ cells/well and incubated for 18 h with 1 × 10^6^ *G. lamblia* trophozoites or Pam_3_CSK_4_ (10 µg/ml) (Invivogen). For kinase inhibition experiment, peritoneal macrophages were pretreated with inhibitors of p38 (SB203580; 30 µM) (Sigma-Aldrich), ERK (PD98059; 40 µM) (Sigma-Aldrich), or AKT (AKT inhibitor IV; 5 µM) (Santa Cruz) for 30 min at 37°C. In TLR2-blocking experiment, WT peritoneal macrophages were pretreated with blocking antibody against TLR2 (30 µg/ml) or mice IgG isotype-matched control antibody (30 µg/ml) (eBioscience, San Diego, CA, USA) at 37°C for 30 min. TLR2-blocking antibody or kinase inhibitor-treated peritoneal macrophages were then cocultured with *G. lamblia* trophozoites at 37°C and 5% CO_2_ for 18 h. All supernatants were collected for ELISA assay. The intestinal fluid was collected as described before (28). The contents were kept frozen at −80°C for ELISA. Cytokines ELISA Ready-SET-Go kits (eBioscience, San Diego, CA, USA) were used to detect IL-12 p40, TNF-α, IFN-γ, and IL-6 levels following the manufacturer’s instructions.

### Western Blot Analysis

Peritoneal macrophages were stimulated under different conditions: non-stimulated macrophages were used as the negative control for phosphorylation of ERK, p38 MAP kinase, p65, and AKT. For Western blot analysis, 2 × 10^6^ macrophages were incubated with 1 × 10^6^ *G. lamblia* trophozoites for 15 min, 30 min, 1 h, 2 h, and 4 h, respectively. Cells were harvested and centrifuged at 12,000 *g* for 30 min at 4°C. The pellets were washed twice with sterile PBS and treated with cell lysis buffer supplemented with proteinase inhibitor mixture and phosphatase inhibitor (Sangon Biotech, Shanghai, China). Protein concentrations were measured using the Bradford protein-quantification assay. 30 µg of sample protein/lane was separated with 10% SDS-PAGE electrophoresis. Proteins were then transferred to polyvinyldifluoride membranes (Millipore, Bedford, MA, USA), blocked with 5% skim milk in TBST for 2 h at room temperature. The membranes were incubated overnight at 4°C with primary Abs, including rabbit anti-ERK, rabbit anti-p38, rabbit anti-AKT, rabbit anti-IKBα, rabbit anti-p65, rabbit anti-phospho-ERK, rabbit anti-phospho-p38, rabbit anti-phospho-AKT, rabbit anti-phospho-IKBα, and rabbit anti-phospho-p65 (Cell Signaling Technology), respectively. After 1 h of washing with TBST, membranes were incubated with secondary HRP conjugated goat anti-rabbit IgG (Cell Signaling Technology, dilution 1/5,000) for 1 h at room temperature and washed three times with TBST. Bands were detected using enhanced chemiluminescence (Vigorous, Beijing, China).

### Real-time PCR Measurement for TLR2

2 × 10^6^ peritoneal macrophages were stimulated for 2 h with 1 × 10^6^ *G. lamblia* trophozoites or Pam_3_CSK_4_ (10 µg/ml). After different treatments, cell culture supernatants were discarded, and the cells were washed twice with sterile PBS. Total RNA was isolated from the cells using the TRIzol Reagent (Invitrogen). First-strand cDNA was synthesized by reverse transcription using the total RNA Transcript II reverse transcriptase (TransGen Biotech Company, Beijing, China). RNA expression levels of the analyzed genes were normalized to the amount of β-actin. All primers were synthesized by Sangon (Shanghai, China), and their sequences were as follows: β-actin, sense: 5′-TGCTGTCCCTGTATGCC TCT-3′, antisense: 5′-GGTCTTTACGGATGTCAACG-3′; TLR2, sense: 5′-CCCACTTCAGGCTCTTTGAC-3′, antisense: 5′-GCCACTCCAGGTAGGTCTTG-3′.

### Subcellular Localization of NF-κB

2 × 10^6^ WT and TLR2^−/−^ mouse peritoneal macrophages were seeded in a well of six-well culture plates with sterile glass coverslips. The cells were stimulated with 1 × 10^6^ *G. lamblia* trophozoites for 0 or 60 min at 37°C and washed twice with sterile PBS. After stimulation, cells were fixed with 4% paraformaldehyde for 15 min at room temperature and washed three times with sterile PBS. Then the cells were incubated overnight in 100 µl permeabilization/wash buffer (0.1% Triton X-100/2% FBS/0.1% azide/PBS) containing rabbit anti-NF-κB (Santa Cruz) at a 1:100 dilution at 4°C. Cells were then washed and incubated with FITC-conjugated goat anti-rabbit IgG (Boster, Wuhan, China) secondary antibody for 1 h at room temperature. The cells were washed, and the coverslips were stained with DAPI at room temperature for 5 min. NF-κB localization was observed using a Zeiss LSM 710 confocal microscope equipped with a 633, 1.4-NA, oil-immersion objective (Carl Zeiss).

### Flow Cytometric Analysis

For flow cytometry, mesenteric lymph nodes (MLNs) were collected in HBSS supplemented with 5% FBS and 25 mM HEPES and strained through a 70 mM nylon membrane. Cells were incubated in HBSS supplemented with 3% FBS and 25 mM HEPES to avoid non-specific binding. Then a total of 1 × 10^6^ cells were stained with anti-CD4-PE (clone RM4-5), anti-CD8-APC (clone 53-6.6), and anti-CD3-PerCP (clone 145-2C11; all from Biolegend) for 1 h at 4°C. The cells were then fixed with 1% paraformaldehyde and analyzed by flow cytometry (BD Biosciences) at 10,000 total events/sample.

### Microscopy, Intestinal Morphometry, and Immunohistochemical Staining

At the time of sacrifice, a 2-cm segment of duodenum (4 cm after the pylorus) was removed and fixed in 10% formalin, transferred to 70% EtOH before paraffin embedding, and then stained with H&E. Villus length and crypt depth measurements were performed only on full-length villi with adjacent crypts, as described by Bejo (29). Rabbit anti-goat TLR2 (eBioscience, San Diego, CA, USA) was used in immunohistochemical staining. Level of expression of TLR2 was evaluated by the method previously described. Briefly, stained tissue samples were graded microscopically as follows: 0, the same as background; 0.5, close to background; 1, well-marked positivity; 1.5, focally enhanced; 2, strong positivity; 2.5, very strong positivity (30).

### Statistical Analysis

GraphPad Prism 5 (GraphPad Software, Inc., CA, USA) was utilized to analyze the data measured by ELISA; SPSS version 19.0 (SPSS, Inc., Chicago, IL, USA) was used for the statistical analysis of other experimental data using one-way analysis of variance, followed by Tukey test. All data were expressed as mean ± SD of triplicate experiments. *p* Values < 0.05 were considered statistically significant.

## Results

### *G. lamblia* Trophozoites Activate TLR2 in WT Mouse Peritoneal Macrophages and Mediate Cytokines Secretion in TLR2^−/−^ and WT Mouse Peritoneal Macrophages

**Figure 1:**
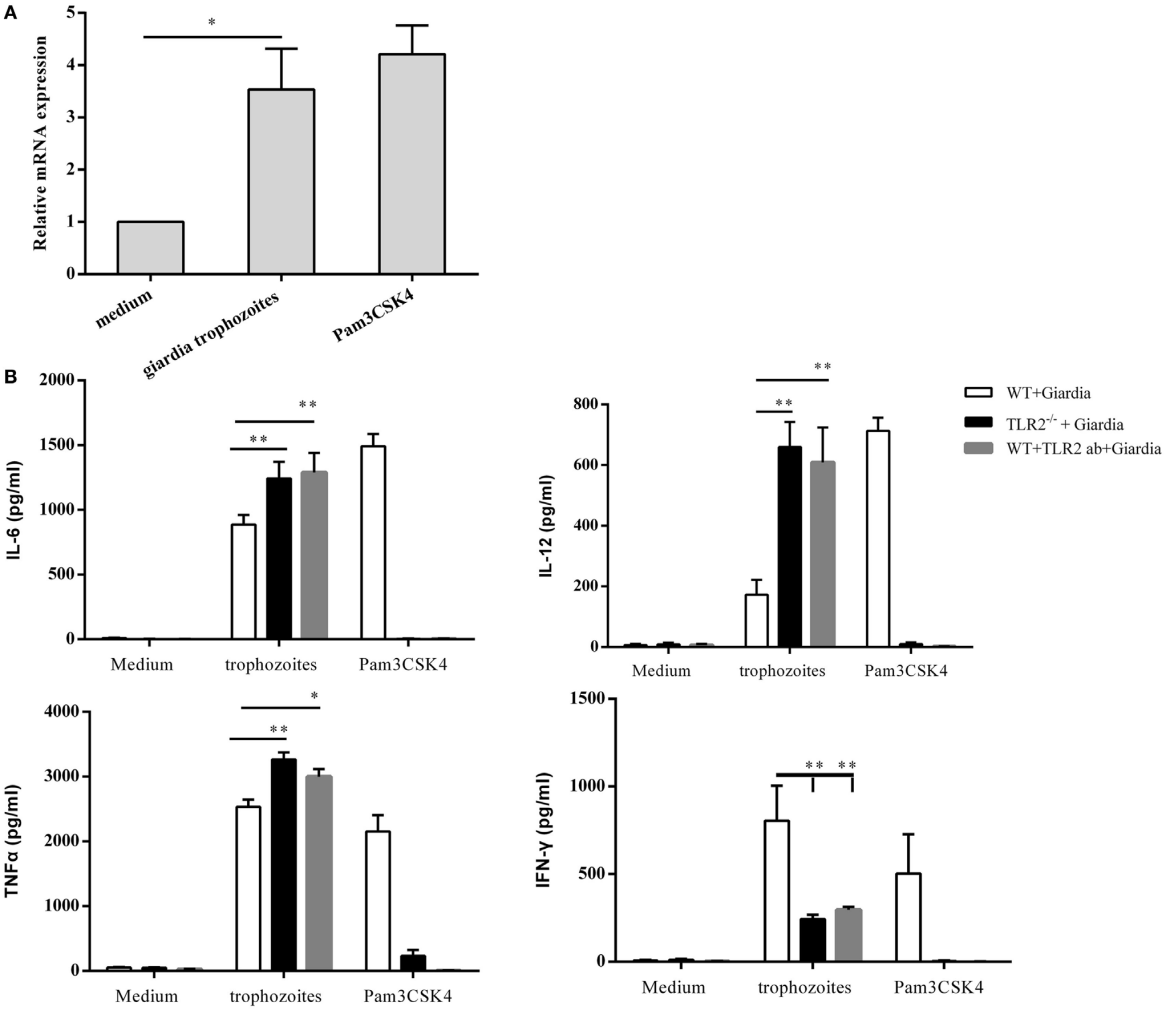
*Giardia lamblia* trophozoites induced cytokines secretion in a TLR2-independent way. RT-PCR analysis of relative mRNA level for TLR2 in total RNA isolated from 2 × 10^6^ mouse peritoneal macrophages incubated in medium alone, with 1 × 10^6^ *G. lamblia* trophozoites, Pam_3_CSK_4_ (10 µg/ml), respectively. The mRNA level was normalized to β-actin **(A)**. TLR2^−/−^ and wild-type (WT) mouse peritoneal macrophages were stimulated with 1 × 10^6^ *G. lamblia* trophozoites or Pam_3_CSK_4_ (10 µg/ml). After 18 h of incubation, the levels of TNF-α, IFN-γ, IL-6, and IL-12 p40 in cell culture supernatant were detected by ELISA **(B)**. Data are expressed as the mean ± SD from three separate experiments. *p < 0.05, **p < 0.01, ***p < 0.001, stimulated cells versus those cultured in medium alone.

Incubation of WT mouse peritoneal macrophages with *G. lamblia* trophozoites for 2 h resulted in a significant increase in TLR2 expression, as compared with negative control cells without stimulation (Figure 1A). To detect the effect of *G. lamblia* trophozoites on the activation of innate immune cells, the production of TNF-α, IFN-γ, IL-6, and IL-12 p40 was investigated in TLR2^−/−^, TLR2-blocked, and WT mouse peritoneal macrophages. Cytokines in the culture supernatants were measured by ELISA after incubation with or without *G. lamblia* trophozoites for 18 h, respectively. We found that TLR2^−/−^ and TLR2-blocked mouse peritoneal macrophages exposed to *G. lamblia* trophozoites produced significantly more TNF-α (*p* < 0.01), IL-6 (*p* < 0.05), and IL-12 p40 (*p* < 0.01) but less IFN-γ (*p* < 0.01) when compared with WT group (Figure 1B).

### *G. lamblia* Trophozoites Induce Cytokines Expression by the Activation of p38 and ERK Pathways *via* TLR2

**Figure 2:**
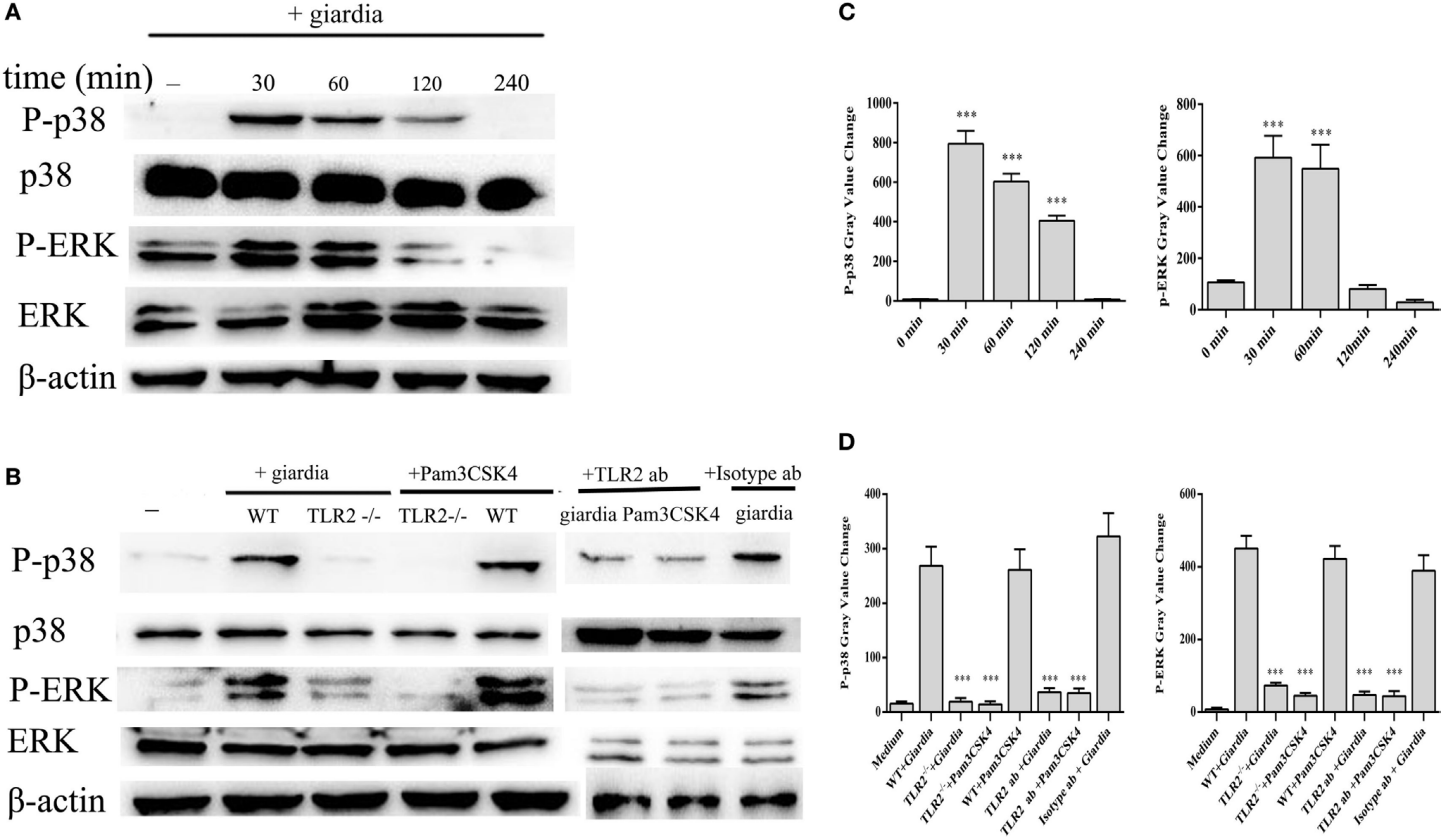
*Giardia lamblia* trophozoites induced the phosphorylation of p38 and ERK *via* TLR2. 2 × 10^6^ wild-type (WT) mouse peritoneal macrophages were stimulated with 1 × 10^6^ *G. lamblia* trophozoites for different times (0–240 min), cell lysates were used for Western blot analysis to measure the levels of phosphorylation of p38 and ERK **(A)**. WT, TLR2^−/−^, and TLR2-blocked mouse peritoneal macrophages were stimulated with *G. lamblia* trophozoites or Pam3CSK4 for 30 min **(B)**. Densitometric analysis of Western blot in panels **(A,B)**, respectively **(C,D)**. Data are expressed as the mean ± SD from three separate experiments. *p < 0.05, **p < 0.01, ***p < 0.001, stimulated cells versus those cultured in medium alone.

*Giardia lamblia* trophozoites-induced activation of MAPKs was detected in peritoneal macrophages with Western blot and phosphor-specific antibodies. *G. lamblia* trophozoites could induce the phosphorylation of p38 and ERK MAP kinases after stimulation with trophozoites for 30 min, while p-JNK was unchanged (data not shown). Phosphorylated p38 peaked at 30 min and returned to baseline at 4 h, while phosphorylated ERK peaked at 30 min and returned to baseline at 2 h. Minimal phosphorylation was observed in negative control cells (Figures 2A,C).

To estimate whether *G. lamblia* trophozoites induced the phosphorylation of p38 and ERK MAP kinases through TLR2, TLR2^−/−^, TLR2 blocked and WT mouse peritoneal macrophages were stimulated with *G. lamblia* trophozoites for 30 min at 37°C. Both TLR2^−/−^ and TLR2-blocked mouse peritoneal macrophages significantly reduced *G. lamblia* trophozoites-induced p38 and ERK phosphorylation. These data suggested that *G. lamblia* trophozoites induced phosphorylation of p38 and ERK MAP kinases *via* TLR2 (Figures 2B,D).

**Figure 3:**
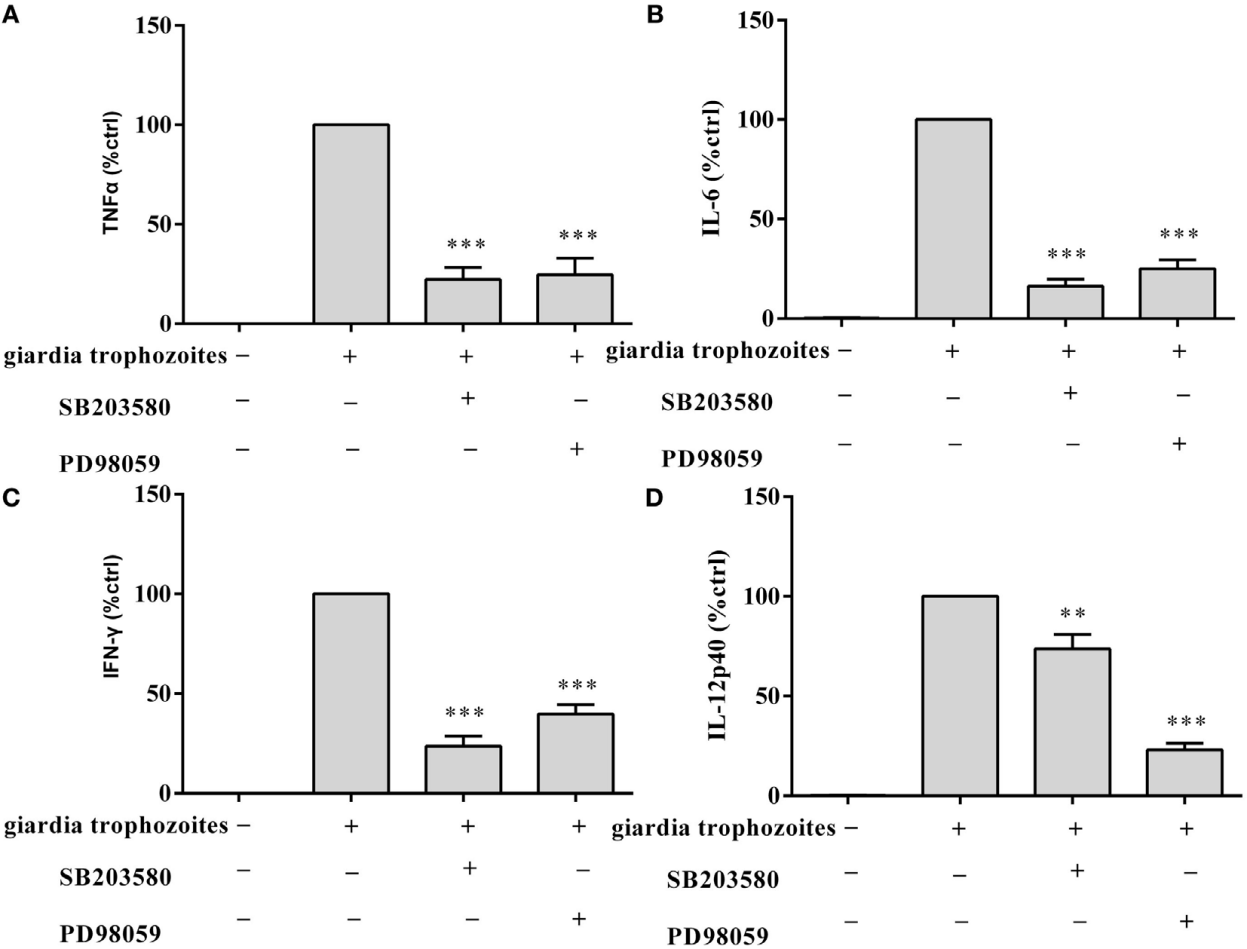
*Giardia lamblia* trophozoites-mediated cytokine expression was blocked by the inhibition of p38 and ERK. 2 × 10^6^ wild-type mouse peritoneal macrophages were pretreated for 60 min with inhibitors of p38 (SB203580; 30 µM) or ERK (PD98059; 40 µM) before stimulation with *G. lamblia* trophozoites. After incubation, the secretion levels of TNF-α, IFN-γ, IL-6, and IL-12 p40 in cell supernatant were measured by ELISA **(A–D)**. The mean ± SD levels of *G. lamblia* trophozoites-induced TNF-α, IFN-γ, IL-6, and IL-12 p40 were set to 100%, and the relative loss of cytokine in the presence of each inhibitor was calculated and expressed as the percentage of control (% ctrl). Data were expressed as the mean ± SD from three separate experiments. *p < 0.05, **p < 0.01, ***p < 0.001, cells treated with *G. lamblia* trophozoites and the inhibitor versus cells stimulated with *G. lamblia* trophozoites alone.

To investigate the specificity of the role of p38 and ERK signaling pathways in the regulation of TNF-α, IFN-γ, IL-6, and IL-12 p40 expression, we used MAPK inhibitors of SB203580 (p38) and PD98059 (ERK). WT peritoneal macrophages were pretreated with or without inhibitors for 30 min at 37°C, and then incubated with *G. lamblia* trophozoites for 18 h. Cytokine levels were measured by ELISA. Both p38 and ERK inhibitors significantly blocked the *G. lamblia*-induced increase in the production of TNF-α (Figure 3A, 557.5 pg/ml *p* < 0.001, 607.7 pg/ml *p* < 0.001), IL-6 (Figure 3B, 138,55 pg/ml *p* < 0.001, 212.85 pg/ml *p* < 0.001), IFN-γ (Figure 3C, 201.45 pg/ml *p* < 0.001, 335.7 pg/ml *p* < 0.001), and IL-12 p40 (Figure 3D, 117.3 pg/ml *p* < 0.01, 41.4 pg/ml *p* < 0.001), respectively.

### *G. lamblia* Trophozoites Reduce Cytokines Secretion by Activating AKT Pathway

**Figure 4:**
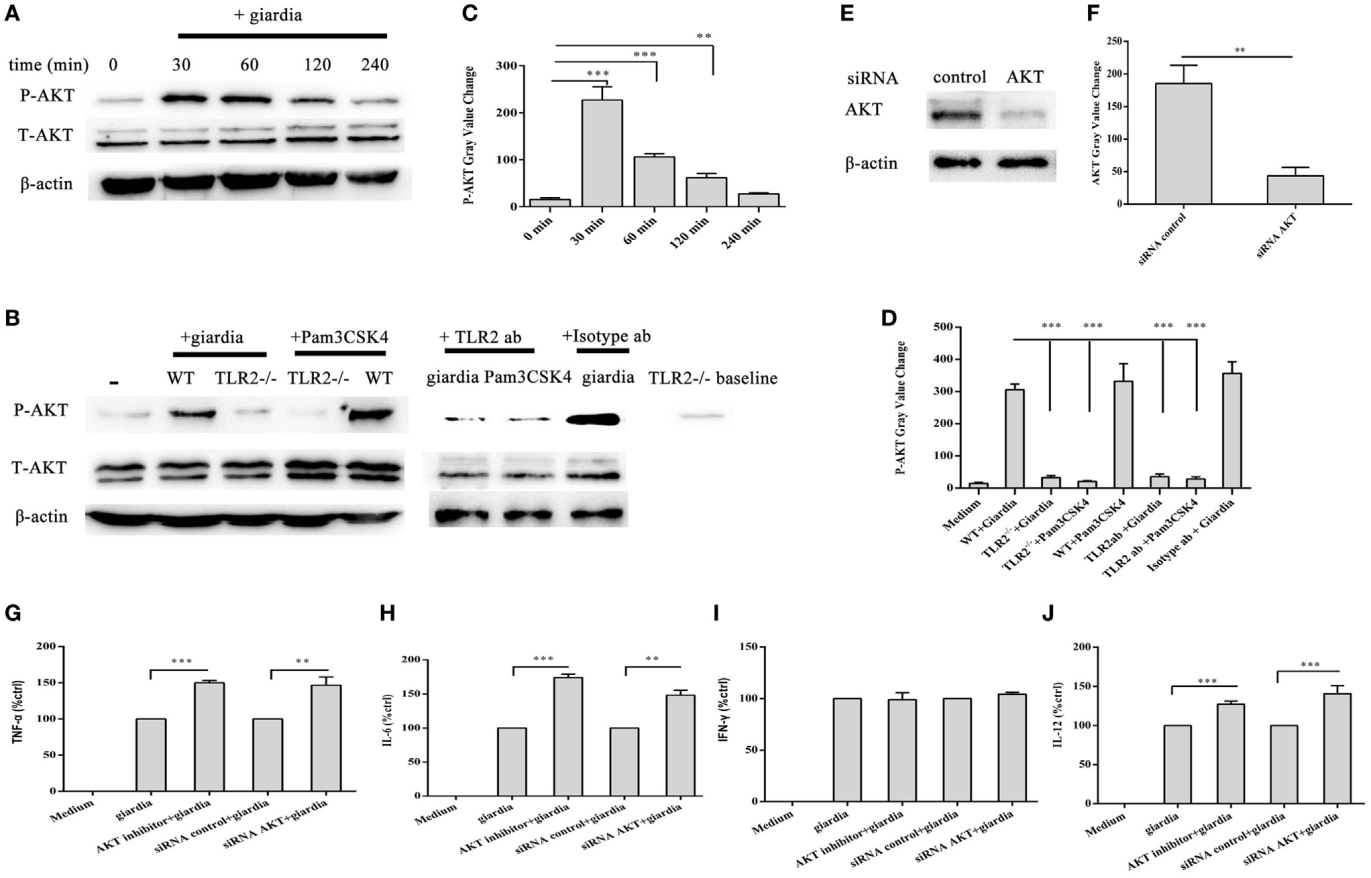
*Giardia lamblia* trophozoites enhanced cytokine expression by inhibition of AKT signal pathway *via* TLR2. 2 × 10^6^ wild-type (WT) mouse peritoneal macrophages were stimulated with 1 × 10^6^ *G. lamblia* trophozoites for different times (0–240 min), cell lysates were used for Western blot analysis to measure the levels of phosphorylation of AKT **(A)**. WT, TLR2-blocked, and TLR2^−/−^ mouse peritoneal macrophages were stimulated with *G. lamblia* trophozoites for 30 min **(B)**. Densitometric analysis of Western blot in panels **(A,B)**, respectively **(C,D)**. 2 × 10^6^ WT mouse peritoneal macrophages were transfected with small-interfering RNA (siRNA)-control or siRNA AKT **(E)**. Densitometric analysis of Western blot in panel **(E) (F)**. 2 × 10^6^ WT mouse peritoneal macrophages were transfected with siRNA AKT or pretreated for 60 min with AKT inhibitor (AKT inhibitor IV) before stimulation with *G. lamblia* trophozoites. After incubation, the secretion levels of TNF-α, IFN-γ, IL-6, and IL-12 p40 in cell supernatant were measured by ELISA **(G–J)**. The mean ± SD levels of *G. lamblia* trophozoites-induced TNF-α, IFN-γ, IL-6, and IL-12 p40 were set to 100%, and the relative loss of cytokine in the presence of AKT inhibitor or siRNA AKT was calculated and expressed as the percentage of control (% ctrl). Data were expressed as the mean ± SD from three separate experiments. *p < 0.05, **p < 0.01, ***p < 0.001, cells treated with *G. lamblia* trophozoites and the inhibitor versus cells stimulated with *G. lamblia* trophozoites alone.

*Giardia lamblia* trophozoites-induced activation of AKT was detected in WT peritoneal macrophages with Western blot and phosphor-specific antibody. *G. lamblia* trophozoites induced the phosphorylation of AKT after stimulation for 30 min. Phosphorylated AKT peaked at 30 min and returned to baseline at 1 h. Minimal phosphorylation was observed in negative control cells (Figures 4A,C). To estimate whether *G. lamblia* trophozoites induced the phosphorylation of AKT through TLR2, TLR2^−/−^, TLR2 blocked, and WT mouse peritoneal macrophages were stimulated with *G. lamblia* trophozoites for 30 min at 37°C. AKT phosphorylation induced by *G. lamblia* trophozoites significantly reduced in both TLR2^−/−^ and TLR2-blocked mouse peritoneal macrophages (Figures 4B,D).

To investigate the specificity of the role of AKT signaling pathway in the regulation of TNF-α, IFN-γ, IL-6, and IL-12 p40 expression, we used AKT inhibitors of AKT inhibitor IV. WT peritoneal macrophages were pretreated with or without AKT inhibitor IV for 30 min at 37°C, and then incubated with *G. lamblia* trophozoites for 18 h. Cytokine levels were measured by ELISA. AKT inhibitor IV significantly enhanced the *G. lamblia*-induced production of TNF-α (Figure 4G, *p* < 0.001), IL-6 (Figure 4H, *p* < 0.001), and IL-12 p40 (Figure 4J, *p* < 0.001), respectively. To confirm this result, WT peritoneal macrophages were transfected with siRNA-control or siRNA AKT for 24 h using Lipofectamine 2000 transfection reagent. Following transfection, cells were furthers stimulated with 1 × 10^6^ *G. lamblia* trophozoites for 18 h. AKT expression levels were detected by Western blot. SiRNA AKT significantly reduced AKT expression (Figures 4E,F). Cytokine levels were measured by ELISA. SiRNA AKT significantly enhanced the *G. lamblia*-induced production of TNF-α (Figure 4G, *p* < 0.05), IL-6 (Figure 4H, *p* < 0.05), and IL-12 p40 (Figure 4J, *p* < 0.001), respectively.

### *G. lamblia* Trophozoites Induce Translocation of NF-κB/rel Subunits to the Nucleus in both TLR2^−/−^ and WT Mouse Peritoneal Macrophages

**Figure 5:**
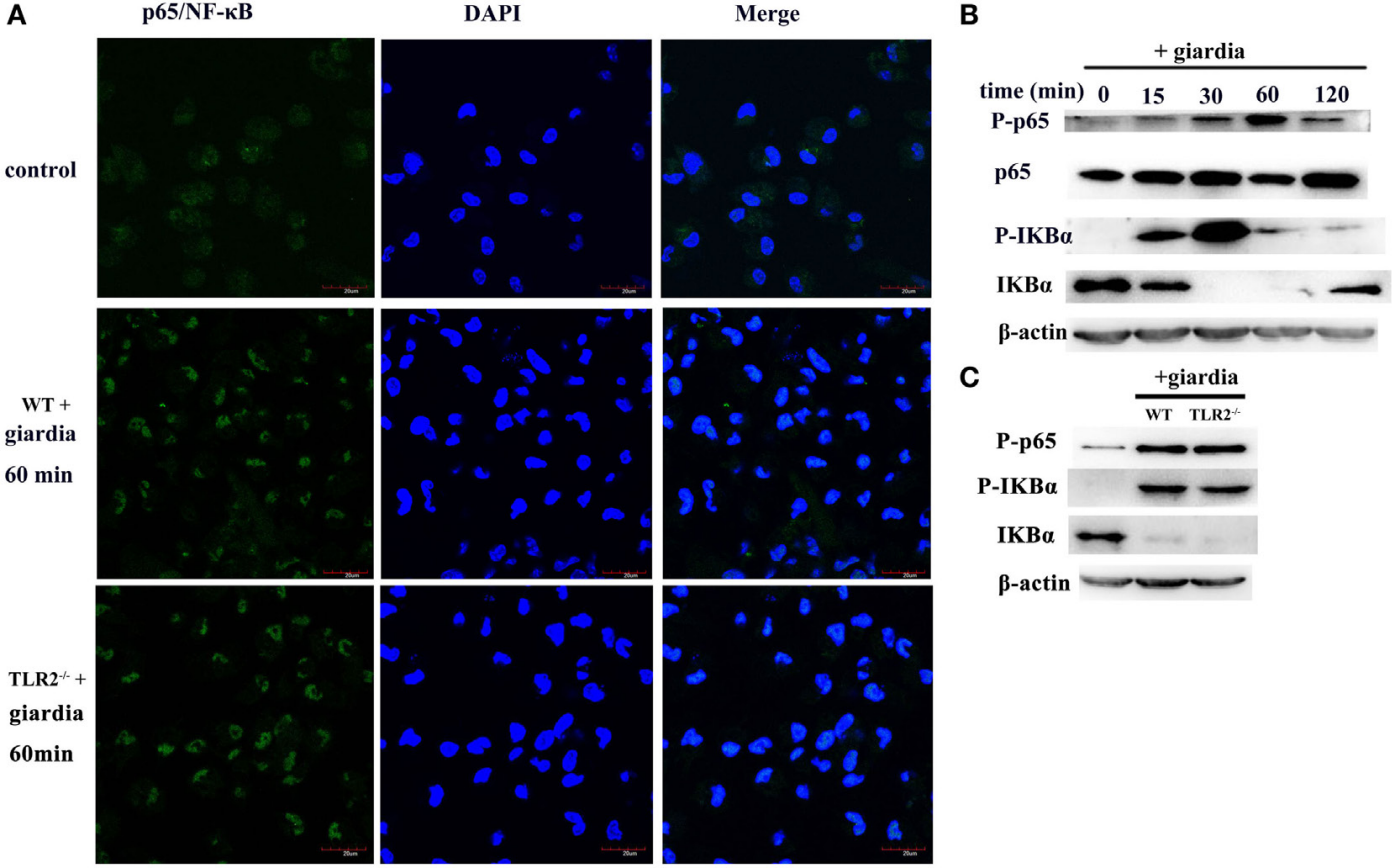
*Giardia lamblia* trophozoites induced changes of subcellular localization of NF-κB/rel subunits. Confocal microscopy [**(A)**, oil-immersion objective] and Western blot analysis **(B,C)** were used to measure the effects of *G. lamblia* trophozoites on the translocation of NF-κB from the cytoplasm to the nucleus in both wild-type (WT) and TLR2^−/−^ mouse peritoneal macrophages. Scale bars: 20 µm **(A)**. 2 × 10^6^ WT mouse peritoneal macrophages were stimulated with 1 × 10^6^ *G. lamblia* trophozoites for different times (0–120 min), cell lysates were used for Western blot analysis. The phosphorylation of p65, IκBα, and total IκBα levels in the cytoplasmic of WT mouse peritoneal macrophages was detected **(B)**. WT and TLR2^−/−^ mouse peritoneal macrophages were stimulated with *G. lamblia* trophozoites for 60 min. The phosphorylation of p65 and IκBα in the cytoplasmic was detected by Western blot **(C)**. Data were acquired by three independent experiments.

The effects of *G. lamblia* trophozoites on NF-κB activation and translocation were detected with immunofluorescence staining and Western blot analysis. Confocal microscopy demonstrated the localization of NF-κB p65 to the nuclei of *G. lamblia* trophozoites-treated cells. After 60 min of stimulation with *G. lamblia* trophozoites, the nuclei were filled with active NF-κB p65 in both TLR2^−/−^ and WT mouse peritoneal macrophages (Figure 5A). In unstimulated cells, NF-κB p65 was located primarily in the cytoplasm (Figure 5A). Western blot analysis also showed that stimulation of WT mouse peritoneal macrophages with *G. lamblia* trophozoites induced the expression of nuclear NF-κB. Nuclear translocation of NF-κB was preceded by degradation of IκBα, the inhibitor of NF-κB (Figure 5B). In addition, the expression level of nuclear NF-κb and IκBα in TLR2^−/−^ mouse macrophages was similar to WT mouse macrophages (Figure 5C).

### TLR2^−/−^ Mice Display Decreased Severity of Giardiasis

**Figure 6:**
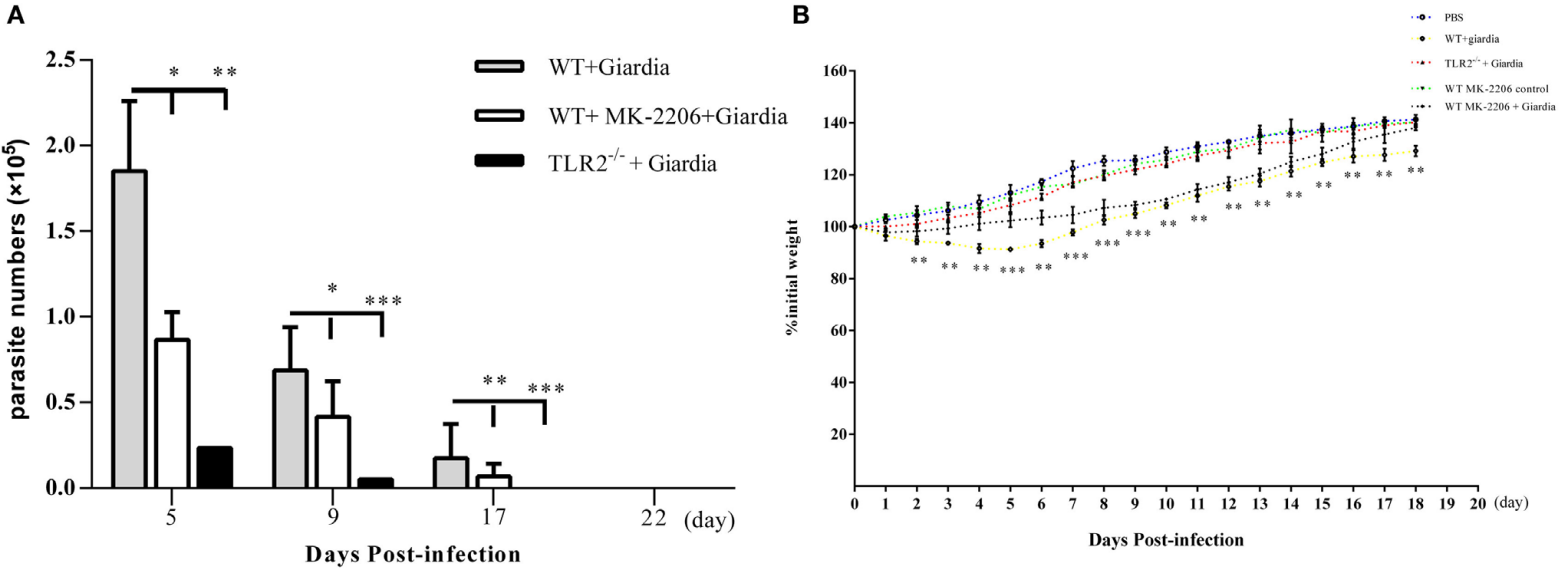
Challenge with *Giardia lamblia* WB cysts in wild-type (WT) mice was longer persistence and caused weight loss in compared with TLR2^−/−^ mice. TLR2^−/−^, AKT-blocked, and WT mice were infected on day 0 with WB strain of *G. lamblia*. Mice were euthanized on the indicated days post-infection, and parasite loads in the small intestine were calculated as described in the Section “Materials and Methods” **(A)**. Growth curves measuring the percentage of change in weight beginning on the day of infection with *G. lamblia* WB cysts (*n* = 10 mice per group) or PBS control (*n* = 10 mice per group) **(B)**. Data were acquired by three independent experiments. *p < 0.05, **p < 0.01, ***p < 0.001, mice challenge with *G. lamblia* WB cysts versus mice challenge with PBS.

**Figure 7:**
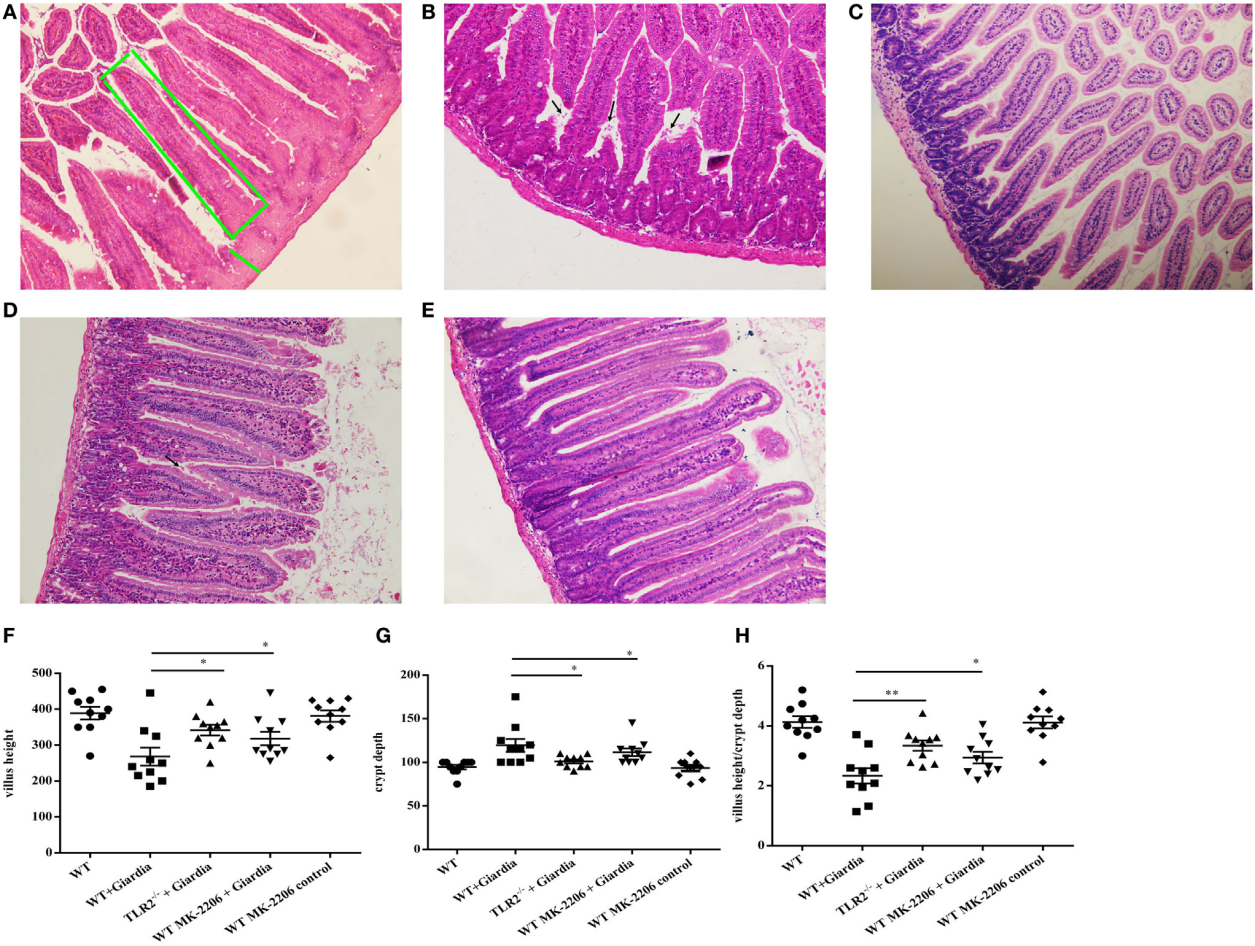
Infected TLR2^−/−^ and AKT-blocked mice challenged with *Giardia lamblia* WB cysts showed lighter lesions intestinal mucosa damage and crypt hyperplasia. H&E-stained duodenum sections from a representative PBS-challenged mouse at 9 dpi. The green box represents villus height. The green line represents crypt depth **(A)**. H&E-stained duodenum sections from a representative *Giardia*-challenged wild-type (WT) mouse at 9 dpi. Black arrows indicate *G. lamblia* **(B)**. H&E-stained duodenum sections from a representative *Giardia*-challenged TLR2^−/−^ mouse at 9 dpi **(C)**. H&E-stained duodenum sections from a representative *Giardia*-challenged AKT-blocked mouse at 9 dpi. Black arrows indicate *G. lamblia* **(D)**. H&E-stained duodenum sections from a representative AKT-blocked *control* mouse at 9 dpi **(E)**. Villus and crypt architectural changes following *G. lamblia* infection in WT and TLR2^−/−^ mice at 9 dpi (*n* = 10 mice per group) **(F–H)**. **(A–C)** Original magnification: ×20. *p < 0.05, **p < 0.01, ***p < 0.001, mice challenge with *G. lamblia* WB cysts versus mice challenge with PBS.

**Figure 8:**
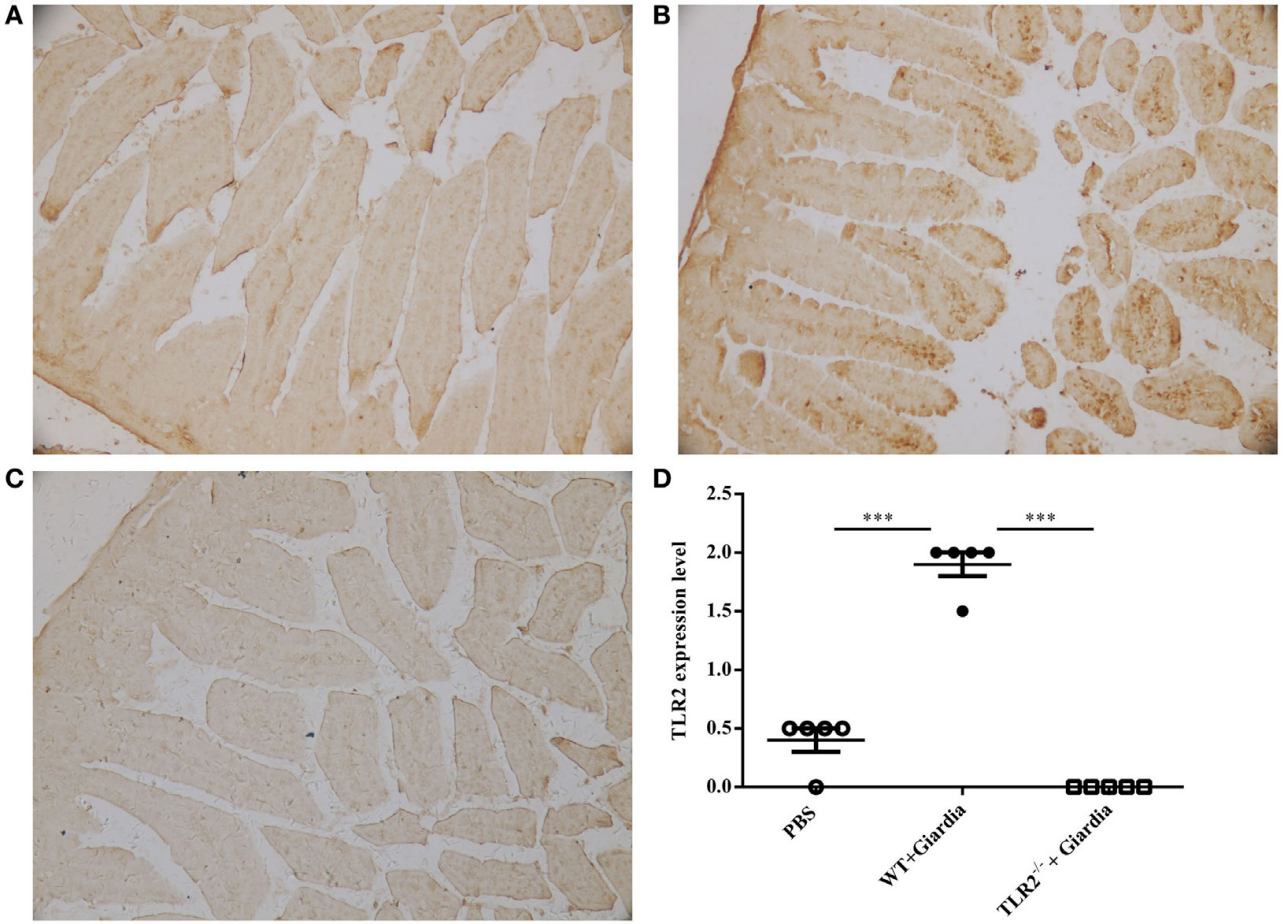
Wild-type (WT) mice challenged with *Giardia lamblia* WB cysts showed activation of TLR2 in intestinal duodenum. Immunohistochemical analysis of duodenum sections from a representative PBS-challenged mouse at 9 dpi with monoclonal TLR2 antibody **(A)**. Immunohistochemical analysis of duodenum sections from a representative *Giardia*-challenged WT mouse at 9 dpi with a monoclonal TLR2 antibody **(B)**. Immunohistochemical analysis of duodenum sections from a representative *Giardia*-challenged TLR2^−/−^ mouse at 9 dpi with a monoclonal TLR2 antibody **(C)**. The degree of expression of TLR2 in the small intestine was measured as described in the Section “Materials and Methods” (*n* = 10 mice per group) **(D)**. Original magnification: ×20. *p < 0.05, **p < 0.01, ***p < 0.001, mice challenge with *G. lamblia* WB cysts versus mice challenge with PBS.

The role of host TLR2 on giardiasis was explored in TLR2^−/−^, AKT-blocked (using MK-2206) and WT mice experimentally infected with *G. lamblia* cysts. The numbers of *G. lamblia* trophozoites were counted at different day post-infection (dpi). No trophozoites were found in PBS control mice (data not shown). Both TLR2^−/−^ and AKT-blocked mice showed significantly less parasite burden and shorter persistence than WT mice during *Giardia* infection (Figure 6A). *Giardia* trophozoites colonization peaked at 5 dpi and disappeared at 22 dpi in both AKT-blocked and WT mice, while peaked at 5 dpi and disappeared at 17 dpi in TLR2^−/−^ mice (Figure 6A). Both TLR2^−/−^ and AKT-blocked mice presented with significantly higher weight gain rate than WT mice during *Giardia* infection (Figure 6B). Significantly higher growth rate was observed in infected TLR2^−/−^ mice from 1 to 18 dpi compared with infected WT mice (*p* < 0.01). The maximum growth difference was observed at 9 dpi (*p* < 0.001), and incomplete recovery was observed at 18 dpi (Figure 6B, *p* < 0.01). Pathological section results suggested that the duodenum of infected TLR2^−/−^ and AKT-blocked mice had minor lesions compared with infected WT mice (Figures 7A–E). Histological morphometry suggested shortened villus length (Figure 7F, *p* < 0.05), hyperplastic crypt (Figure 7G, *p* < 0.05), and decreased ratio of villus height/crypt depth (Figure 7H, *p* < 0.01) in infected WT mice compared with TLR2^−/−^ and AKT-blocked mice. Expanded lamina propria was observed in TLR2^−/−^, AKT-blocked, and infected WT mice compared with PBS control mice (Figures 7A–E). As shown in Figure 8D, *G. lamblia* was associated with increased TLR2 antibody staining in the villus in infected WT mice (Figure 8B) compared with infected TLR2^−/−^ mice (Figure 8C) and PBS control mice (Figure 8A) (*p* < 0.001). Moreover, TLR2 expression was more apparent in the villus surface epithelium than that in the depth of crypts (Figure 8B).

### TLR2^−/−^ Mice Showed Higher Percentage of CD4^+^ T Cell Population and Elevated Cytokine Production during *G. lamblia* Infection

**Figure 9:**
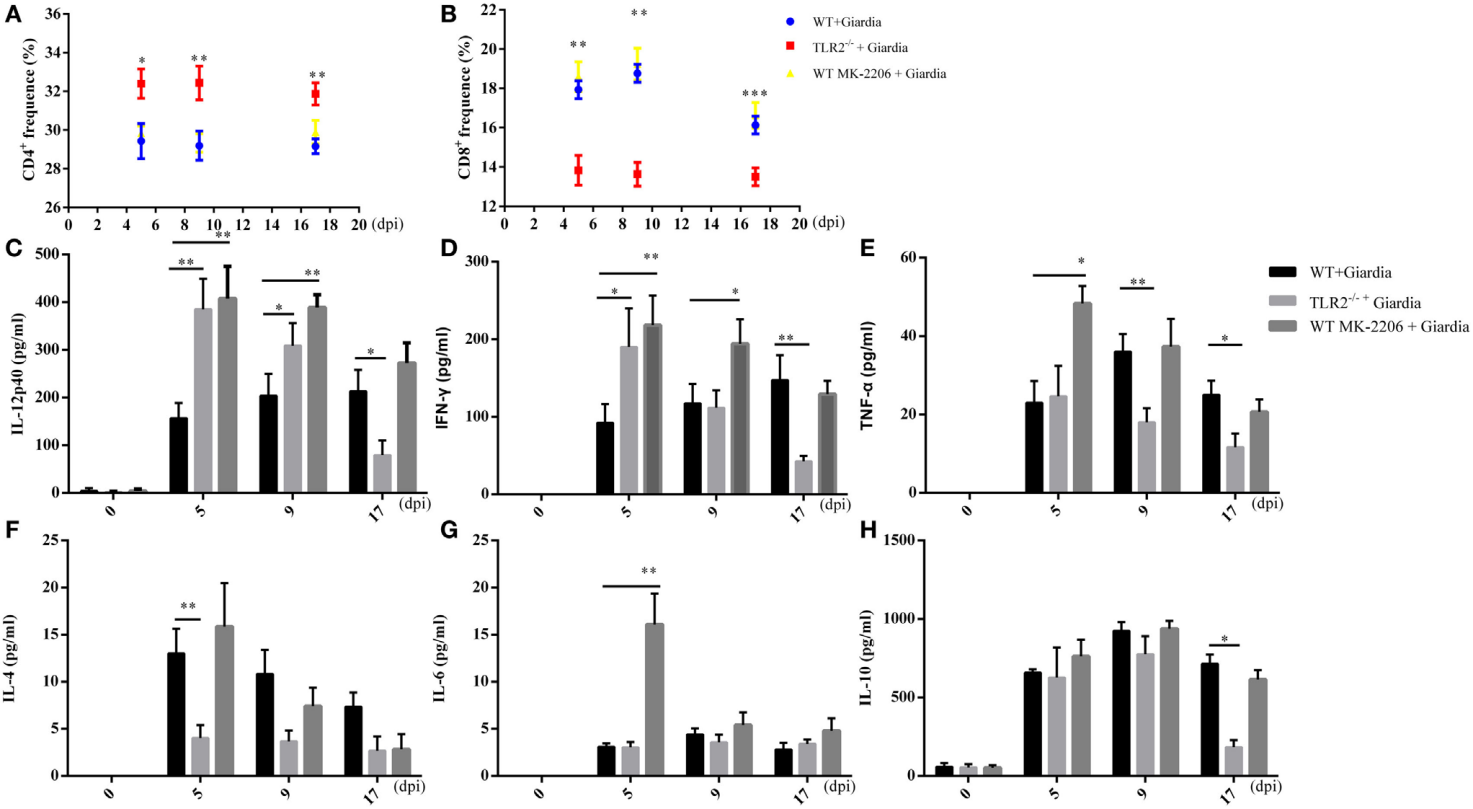
Cytokines production in the small intestine of wild-type (WT), AKT-blocked, and TLR2^−/−^ mice infected with *Giardia lamblia*. WT, AKT-blocked, and TLR2^−/−^ mice were infected with *G. lamblia* and euthanized at 5, 9, and 17 dpi. The proportions of CD4^+^ **(A)** and CD8^+^ **(B)** cells in mesenteric lymph nodes were detected during the course of infection. Production of IL-12 **(C)**, IFN-γ **(D)**, TNF-α **(E)**, IL-4 **(F)**, IL-6 **(G)**, and IL-10 **(H)** in small intestine were measured using ELISA. *p < 0.05, **p < 0.01, ***p < 0.001, mice challenge with *G. lamblia* WB cysts versus mice challenge with PBS. Data presented are means and SEM for 10 mice per time point.

Flow cytometry analysis indicated that the percentage of CD4^+^ T cell population was significantly higher during the infection in MLNs of infected TLR2^−/−^ mice compared with that of infected AKT-blocked and WT mice (Figure 9A), while the percentage of CD8^+^ T cell population was significantly lower in MLNs of infected TLR2^−/−^ mice compared with that of infected AKT-blocked and WT mice (Figure 9B).

To explore whether the deficiency of TLR2 results in adaptive immune response in infected mice, the intestinal fluids were collected following sacrifice of the mice. Our results showed the intestinal fluids of TLR2^−/−^ mice produced significantly more IL-12 p40 (Figure 9C) and IFN-γ (Figure 9D), while significantly less IL-4 was produced during the early stage of infection (5 dpi) (Figure 9F) compared with WT mice. In addition, the production of TNF-α, IL-6, and IL-10 was similar between TLR2^−/−^ and WT mice during the early stage of infection (5 dpi) (Figures 9E,G,H).

To explore whether the blocking of AKT results in adaptive immune response in infected mice, the intestinal fluids were collected following sacrifice of the mice. Our results showed the intestinal fluids of AKT-blocked mice produced significantly more IL-12 p40 (Figure 9C), IFN-γ (Figure 9D), TNF-α (Figure 9E), and IL-6 (Figure 9F) during the early stage of infection (5 dpi) compared with infected WT mice. In addition, the production of IL-10 was similar between AKT-blocked and WT mice during the early stage of infection (5 dpi) (Figure 9H).

## Discussion

Previous studies have demonstrated that DCs, mast cells, macrophages, and intestinal epithelial cells are involved in parasites control (21, 31, 32). Macrophages can ingest *G. lamblia* trophozoites and accumulate in the lamina propria after infection (32, 33), which indicated that macrophages may play an important role in the clearance of *Giardia* infection. *Giardia* trophozoites lysate may interfere with the activation of DC through TLR2 *in vitro* (20). Our results showed that *G. lamblia* trophozoites enhanced the expression of TLR2 in WT mouse peritoneal macrophages.

To explore the role of TLR2 during *G. lamblia* infection *in vivo*, we developed a mouse model of persistent giardiasis using purified *G. lamblia* WB cysts. We found that *Giardia* trophozoites colonization peaked at 5 dpi and disappeared at 22 dpi in WT mice. Previous *in vivo* studies have indicated that *Giardia* trophozoites colonization peaked at 5 dpi (31), which is consistent with the present results. In addition, histological morphometry suggested shortened villus length, hyperplastic crypt, and decreased ratio of villus height/crypt depth in infected WT mice, which was consistent with previous studies in human duodenum (34). To the best our knowledge, this is the first mouse giardiasis model using purified *G. lamblia* WB cysts, which are induced from axenic trophozoites *in vitro*. Compared with mouse model infected by trophozoites, mouse model infected by *G. lamblia* cysts might be more similar to natural infection. Most important of all, it is the first time that we found that *G. lamblia* activated duodenum TLR2 in the mouse model. Our study demonstrated that TLR2 could be activated during *G. lamblia* stimulation *in vitro* or infection *in vivo* and it may play an important role on defending *G. lamblia* infection.

Pro-inflammatory cytokines such as TNF-α and IL-6 were necessary for effective *Giardia* control in mice (35–37). TNF-α-deficient mice do not appear to be related to mechanisms previously shown to control *Giardia* infections, including IgA production, mast cell responses, IL-6 or IL-4 expression (37). IL-6 has been found to be necessary for the clearance of *Giardia* in a mouse infection model. In contrast to WT mice, IL-6-deficient mice were not able to control *Giardia* infection. Furthermore, mast cells are also involved in the control of *Giardia* infection through IL-6 production (35, 36, 38). *Giardia* induce inflammatory responses, with the involvement of blood platelets and release of IFN-γ, TNF-α, and IL-6 (39). Analysis of cytokines production by spleen and MLN cells in mice model have shown the production of IL-4, IL-10, IL-13, IL-17, IL-22, TNF-α, and IFN-γ after infection with both WB and GS strains (26). Activation of TLRs could initiate a range of host defense mechanisms, such as the activation of NF-κB and production of pro-inflammatory cytokines that contribute to the effective elimination of pathogenic microorganisms (11). Studies also suggested that co-incubation of bone marrow-derived DCs with *Giardia* extracts and TLR ligands leads to upregulation of IL-10 and downregulation of IL-12 (20). TLR2 ligand-stimulated DCs incubated in the presence of *Giardia* trophozoites lysate produced less IL-12/23P40, il-12P70, and IL-23, but more IL-10 than cells incubated without the parasite (21). Interestingly, our study demonstrated that the capacity to induce TNF-α, IL-6, and IL-12 p40 was enhanced in TLR2^−/−^ mouse macrophages compared with WT mouse macrophages, indicating that TLR2 played an inhibit role in *G. lamblia* trophozoites-induced cytokines production. However, the expression of IL-12 p40 in our study was different from previous data (20), which might be due to the different choices of *G. lamblia* trophozoites, mouse peritoneal macrophages, or the ratio between trophozoites and macrophages. Infected TLR2^−/−^ mice showed enhanced production of IL-12 p40 and IFN-γ compared with infected WT mice, as demonstrated by ELISA at the early stage (5 dpi) during infection, while infected AKT-blocked mice showed enhanced production of IL-12 p40, IFN-γ, IL-6, and TNF-α compared with infected WT mice. We found that TLR2^−/−^ mice did not affect TNF-α and IL-6 production *in vivo*, while AKT-blocked mice did increase the production of these two cytokines during *Giardia* infection. In addition, macrophages from TLR2^−/−^ mice *in vitro* showed enhanced production of IL-12 p40, TNF-α, and IL-6 but not IFN-γ, while TLR2^−/−^ mice only showed enhanced production of IL-12 p40 and IFN-γ *in vivo* in response to *Giardia* infection. AKT-blocked macrophage *in vitro* enhanced the production of IL-12 p40, TNF-α, and IL-6 but not IFN-γ, while the AKT inhibitor still enhanced IFN-γ production *in vivo* in response to *Giardia* infection. Evidently, the *in vivo* results of cytokine production using TLR2^−/−^ mice and AKT inhibitor did not match completely with *in vitro* results. One possible explanation for this discrepancy could be that macrophages are not the only TLR2-expressing cells involved during *Giardia* infection *in vivo*. Previous studies have demonstrated that macrophage activity represents intraepithelial antigen processing as well as defense against the effects of the uncontrolled entrance of microorganisms and other antigenic particles into Peyer’s patch lymphoid follicles, and macrophages are capable of ingesting *G. lamblia in vitro* and may play an important role in host defense in giardiasis (33, 40, 41). Adoptive transfer of DCs loaded with *Giardia* antigens led to reduced infection intensity in both wild-type (WT)- and IL-6-deficient mice. Thus, the limited activation of DCs by *Giardia* is sufficient to induce protective responses. Moreover, defects in IL-6 knockout mice can be traced to the development and/or function of DCs. These studies suggest that DCs have crucial roles in anti-*Giardia* immunity (20, 42–44). In addition, mast cells are also recruited following infection and are required for the efficient control of infection (31, 38).

The MAPK signal pathway controls gene expression and immune function and mediates the regulation of proinflammatory cytokine production (45). Parasite GPI-induced cellular activation is mediated mainly by TLR2, initiating the MAPK and NF-KB signal pathways (46). *G. lamblia* GS ESPs can trigger IL-8 production in HT-29 cells by activating p38 and ERK1/2 signal pathways (47). For the first time our study showed that *G. lamblia* trophozoites activated TLR2, which resulted in the phosphorylation of p38 and ERK MAP kinases and the production of pro-inflammatory cytokines in WT mouse peritoneal macrophages. Furthermore, *G. lamblia* trophozoites-induced production of TNF-α, IFN-γ, IL-6, and IL-12 p40 was significantly decreased by ERK and p38 inhibitors. These data suggested that TLR2-mediated activation of p38 and ERK signal pathways, which played an important role in *G. lamblia* trophozoites-induced production of TNF-α, IFN-γ, IL-6, and IL-12 p40 by mouse peritoneal macrophages. Likewise, p38 and ERK phosphorylation were inhibited significantly in TLR2^−/−^ mouse peritoneal macrophages after stimulated with *G. lamblia* trophozoites, which demonstrated that *G. lamblia* trophozoites mainly induced phosphorylation of p38 and ERK MAP kinases *via* TLR2 in mouse peritoneal macrophages.

AKT signal pathway is activated by a combination of ligands such as lipopolysaccharide (LPS), and cell surface receptors such as TLRs, and various cytokine receptors (48). The AKT signal pathway has been shown to induce a negative regulation of TLR-induced IL-12 and IL-10 secretion, as well as other inflammatory cytokines (48–50). In this study, blocking AKT signal pathway leads to significant increase in cytokine productions including TNF-α, IL-6, and IL-12 p40 in WT mouse macrophages after stimulation with *G. lamblia* trophozoites, while the production of IFN-γ is not affected (Figure 4I). This result suggested that the production of IFN-γ was regulated by p38 and ERK MAP kinases pathway *via* TLR2, rather than AKT pathway. Also *G. lamblia* trophozoites enhanced IL-12 p40, TNF-α, and IL-6 in TLR2^−/−^ mouse macrophages compared with in WT mouse macrophages and increased IL-12 p40 and IFN-γ production *in vivo* in infected TLR2^−/−^ mice. However, the expression of IL-12 p40, IFN-γ, TNF-α, and IL-6 is enhanced *in vivo* in infected AKT-blocked mice compared with infected WT mice. These results suggest that *G. lamblia* trophozoites could regulate the production of cytokines dependent on AKT signal pathway *via* TLR2. Previous study has found that inhibition of the AKT signal pathway could enhance the expression level of IL-12 by *Porphyromonas gingivalis* LPS (51), which is consistent with the current results. Interestingly, the expression level of IL-12 p40 and IFN-γ in infected TLR2^−/−^ mice is similar with that in infected AKT-blocked mice, while the expression level of TNF-α and IL-6 is less in infected TLR2^−/−^ mice than in infected AKT-blocked mice. These results suggest that different immune cells are involved in the clearance of *Giardia* between infected TLR2^−/−^ and AKT-blocked mice. Previous studies have demonstrated that CD4^+^ T cells are required for *Giardia* elimination, whereas CD8^+^ T cells are not required (52), which is consistent with our results. Our results suggested that the numbers of CD4^+^ T cells in infected TLR2^−/−^ mice were increased, while the numbers of CD8^+^ T cells in infected TLR2^−/−^ mice were decreased compared with infected AKT-blocked and WT mice, suggesting that different cytokines might be involved in the clearance of *giardia* in infected TLR2^−/−^ mice compared with *in vitro* experiment data.

NF-κB is the primary transcription factor activated by TLR signaling, which is the key for triggering and coordinating both innate and adaptive immune responses (14). Previous studies have shown that the phosphorylation of NF-κB can be mediated by TLR2 (27), and induction of the NF-κB signaling pathway was correlated with NF-κB p65 (53). Recent studies indicated that a shift from p50–p65 heterodimer to p50 homodimers in NF-κB may participate in resolving inflammation (14, 54). The dynamic balance between cytosolic and nuclear localization of NF-κB is mediated by the degradation of IκBα (55). Data from our study indicated that, in both TLR2^−/−^ and WT mouse macrophages, the level of IκBα in the cytoplasm was reduced and NF-κB p65 was accumulated in the nucleus stimulated by *G. lamblia* trophozoites, suggesting that *G. lamblia* trophozoites activated NF-κB signaling pathway which in turn lead to the increased cytokines production in both TLR2^−/−^ and WT mouse macrophages.

Commonly TLR2 plays a crucial role in pathogen recognition, activation of innate immunity, and pathogen elimination. TLR2 also participates in the host defense against parasite infection such as *T. gondii* and *T. cruzi* (10, 17, 18). However, TLR2^−/−^ mice display increased clearance of Dermatophyte *Trichophyton mentagrophytes* in the setting of hyperglycemia (19). In this study, we found that *Giardia* infection led to a decreased parasite burden, short parasite persistence and an increased weight gain rate in infected TLR2^−/−^ mice compared with in infected WT mice. Histological morphometry suggested shortened villus length, hyperplastic crypt, and decreased ratio of villus height/crypt depth in infected WT mice compared with in TLR2^−/−^ mice. Interestingly, our studies suggest that the weight of infected mouse was decreased from the first day post-infection to the ninth day post-infection, and then increased gradually in infected WT mice compared with infected TLR2^−/−^ mice and PBS control mice, which is different from previous results (56). The difference might be caused by using purified *G. lamblia* WB cysts rather than trophozoites. Intestinal macrophages shape the host immune response to infection, yet we still know little about how these cells respond to *G. lamblia* infection. In this study, we explore the role of TLR2 and AKT signal pathway in macrophages *in vitro*. Our data demonstrate that *giardia* trophozoites can reduce the production of macrophage cytokines through TLR2–AKT signal pathway *in vitro*. Further work should aim to better characterize host macrophage innate immune responses during *G. lamblia* infection.

In summary, a new role for host TLR2 in controlling giardiasis severity has been identified. Our study demonstrated that *G. lamblia* induced a decreased production of proinflammatory cytokines by activating AKT signal pathway *via* TLR2 *in vitro* and which might result in severity giardiasis *in vivo*. On contrast TLR2^−/−^ mice display decreased the severity of giardiasis through enhanced proinflammatory cytokines production. The present results would promote our understanding of molecular mechanism governing the host immune responses against *G. lamblia* infection and help us to design better strategies for *G. lamblia* control.

## Ethics Statement

All animal experimental procedures were performed in strict accordance with the Regulations for the Administration of Affairs Concerning Experimental Animals approved through the State Council of People’s Republic of China (1988.11.1) and with approval of the Animal Welfare and Research Ethics Committee at Jilin University (IACUC Permit Number: 20160612).

## Author Contributions

XL, PG, XZ, and JL drafted the main manuscript and performed the data analysis; XL, FX, and LL planned and performed experiments; XL, PG, and JL were responsible for experimental design; and XZ, ZY, and JL responsible for guiding and supporting the experiments and manuscript revisions.

## Conflict of Interest Statement

The authors declare that the research was conducted in the absence of any commercial or financial relationships that could be construed as a potential conflict of interest.

## References

[1] Savioli L Smith H Thompson A. *Giardia* and *Cryptosporidium* join the ‘Neglected Diseases Initiative’. Trends Parasitol (2006) 22:203–8. 10.1016/j.pt.2006.02.015 16545611

[2] Geurden T Vercruysse J Claerebout E. Is *Giardia* a significant pathogen in production animals? Exp Parasitol (2010) 124:98–106. 10.1016/j.exppara.2009.03.001 19285075

[3] Adam RD Biology of *Giardia lamblia*. Clin Microbiol Rev (2001) 14:447–75. 10.1128/CMR.14.3.447-475.2001 11432808 PMC88984

[4] Thompson RCA Reynoldson JA Mendis AHW *Giardia* and giardiasis. Adv Parasitol (1993) 32:71–160. 10.1016/s0065-308x(08)60207-9 8237618

[5] Thompson RCA. The zoonotic significance and molecular epidemiology of *Giardia* and giardiasis. Vet Parasitol (2004) 126:15. 10.1016/j.vetpar.2004.09.008 15567577

[6] Flanagan PA *Giardia* – diagnosis, clinical course and epidemiology. A review. Epidemiol Infect (1992) 109:1–22. 1499664 PMC2272232

[7] Eckmann L. Mucosal defences against *Giardia*. Parasite Immunol (2003) 25:259–70. 10.1046/j.1365-3024.2003.00634.x 12969444

[8] Buret AG Amat CB Manko A Beatty JK Halliez MCM Bhargava A *Giardia duodenalis*: new research developments in pathophysiology, pathogenesis, and virulence factors. Curr Trop Med Rep (2015) 2:110–8. 10.1007/s40475-015-0049-8

[9] Kawai T Akira S. Pathogen recognition with toll-like receptors. Curr Opin Immunol (2005) 17:338–44. 10.1016/j.coi.2005.02.007 15950447

[10] Oliveira-Nascimento L Massari P Wetzler LM The role of TLR2 in infection and immunity. Front Immunol (2012) 3:79 10.3389/fimmu.2012.00079 22566960 PMC3342043

[11] Takeda K Kaisho T Akira S. Toll-like receptors. Annu Rev Immunol (2003) 21(1):335; Innate immunity signal transduction Myd88 microbial components *Drosophila*. 10.1146/annurev.immunol.21.120601.141126 12524386

[12] Vasselon T Hanlon WA Wright SD Detmers PA. Toll-like receptor 2 (TLR2) mediates activation of stress-activated MAP kinase p38. J Leukoc Biol (2002) 71:503–10. 11867688

[13] Wang Q Dziarski R Kirschning CJ Muzio M Gupta D Micrococci and peptidoglycan activate TLR2 – >MyD88 – >IRAK – >TRAF – >NIK – >IKK – >NF-kappaB signal transduction pathway that induces transcription of interleukin-8. Infect Immun (2001) 69:2270–6. 10.1128/IAI.69.4.2270-2276.2001 11254583 PMC98155

[14] Ashall L Horton CA Nelson DE Paszek P Harper CV Sillitoe K Pulsatile stimulation determines timing and specificity of NF-kappa B-dependent transcription. Science (2009) 324:242–6. 10.1126/science.1164860 19359585 PMC2785900

[15] Soilleux EJ Morris LS Leslie G Chehimi J Luo Q Levroney E Constitutive and induced expression of DC-SIGN on dendritic cell and macrophage subpopulations in situ and in vitro. J Leukoc Biol (2002) 71:445–57. 11867682

[16] Johnson GL Lapadat R. Mitogen-activated protein kinase pathways mediated by ERK, JNK, and p38 protein kinases. Science (2002) 298:1911–2. 10.1126/science.1072682 12471242

[17] Debierre-Grockiego F Campos MA Azzouz N Schmidt J Bieker U Resende MG Activation of TLR2 and TLR4 by glycosylphosphatidylinositols derived from *Toxoplasma gondii*. J Immunol (2007) 179:1129–37. 10.4049/jimmunol.179.2.1129 17617606

[18] Campos MAS Almeida IC Takeuchi O Akira S Valente EP Procopio DO Activation of toll-like receptor-2 by glycosylphosphatidylinositol anchors from a protozoan parasite. J Immunol (2001) 167:416–23. 10.4049/jimmunol.167.1.416 11418678

[19] Almeida DF Fraga-Silva TF Santos AR Finato AC Marchetti CM Golim MA TLR2-/- mice display increased clearance of dermatophyte trichophyton mentagrophytes in the setting of hyperglycemia. Front Cell Infect Microbiol (2017) 7:8. 10.3389/fcimb.2017.00008 28164040 PMC5248405

[20] Kamda JD Singer SM. Phosphoinositide 3-kinase-dependent inhibition of dendritic cell interleukin-12 production by *Giardia lamblia*. Infect Immun (2009) 77:685–93. 10.1128/IAI.00718-08 19047410 PMC2632045

[21] Obendorf J Renner Viveros P Fehlings M Klotz C Aebischer T Ignatius R Increased expression of CD25, CD83, and CD86, and secretion of IL-12, IL-23, and IL-10 by human dendritic cells incubated in the presence of toll-like receptor 2 ligands and *Giardia duodenalis*. Parasit Vectors (2013) 6:317 10.1186/1756-3305-6-317 24499474 PMC4029533

[22] Emery SJ Mirzaei M Vuong D Pascovici D Chick JM Lacey E Induction of virulence factors in *Giardia duodenalis* independent of host attachment. Sci Rep (2016) 6:20765. 10.1038/srep20765 26867958 PMC4751611

[23] Kane AV Ward HD Keusch GT Pereira ME. In vitro encystation of *Giardia lamblia*: large-scale production of in vitro cysts and strain and clone differences in encystation efficiency. J Parasitol (1991) 77:974–81. 10.2307/3282752 1779302

[24] Wang Q Yu WN Chen X Peng XD Jeon SM Birnbaum MJ Spontaneous hepatocellular carcinoma after the combined deletion of Akt isoforms. Cancer Cell (2016) 29:523–35. 10.1016/j.ccell.2016.02.008 26996309 PMC4921241

[25] Cheng Y Ren X Zhang Y Patel R Sharma A Wu H eEF-2 kinase dictates cross-talk between autophagy and apoptosis induced by Akt Inhibition, thereby modulating cytotoxicity of novel Akt inhibitor MK-2206. Cancer Res (2011) 71:2654–63. 10.1158/0008-5472.CAN-10-2889 21307130 PMC3210447

[26] Solaymani-Mohammadi S Singer SM. Host immunity and pathogen strain contribute to intestinal disaccharidase impairment following gut infection. J Immunol (2011) 187:3769–75. 10.4049/jimmunol.1100606 21873528 PMC3178714

[27] Chen ST Li JY Zhang Y Gao X Cai H. Recombinant MPT83 derived from *Mycobacterium tuberculosis* induces cytokine production and upregulates the function of mouse macrophages through TLR2. J Immunol (2012) 188:668–77. 10.4049/jimmunol.1102177 22174456

[28] Goyal N Shukla G. Probiotic *Lactobacillus rhamnosus* GG modulates the mucosal immune response in *Giardia intestinalis*-infected BALB/c mice. Dig Dis Sci (2013) 58:1218–25. 10.1007/s10620-012-2503-y 23263901

[29] Bejo MH Gastrointestinal Response to Copper Excess: Studies on Copper (and Zinc) Loader Rats [PhD Thesis] UK: University of Liverpool (1990).

[30] Frolova L Drastich P Rossmann P Klimesova K Tlaskalovahogenova H. Expression of toll-like receptor 2 (TLR2), TLR4, and CD14 in biopsy samples of patients with inflammatory bowel diseases: upregulated expression of TLR2 in terminal ileum of patients with ulcerative colitis. J Histochem Cytochem (2008) 56:267–74. 10.1369/jhc.7A7303.2007 18040078 PMC2324181

[31] Li E Tako EA Singer SM. Complement activation by *Giardia duodenalis* parasites through the lectin pathway contributes to mast cell responses and parasite control. Infect Immun (2016) 84:1092–9. 10.1128/IAI.00074-16 26831470 PMC4807472

[32] Maloney J Keselman A Li E Singer SM. Macrophages expressing arginase 1 and nitric oxide synthase 2 accumulate in the small intestine during *Giardia lamblia* infection. Microbes Infect (2015) 17:462–7. 10.1016/j.micinf.2015.03.006 25797399 PMC4461514

[33] Belosevic M Daniels CW. Phagocytosis of *Giardia lamblia* trophozoites by cytokine-activated macrophages. Clin Exp Immunol (1992) 87:304–9. 10.1111/j.1365-2249.1992.tb02992.x 1735194 PMC1554267

[34] Troeger H Epple HJ Schneider T Wahnschaffe U Ullrich R Burchard GD Effect of chronic *Giardia lamblia* infection on epithelial transport and barrier function in human duodenum. Gut (2007) 56:328–35. 10.1136/gut.2006.100198 16935925 PMC1856804

[35] Zhou P Li E Zhu N Robertson J Nash T Singer SM. Role of interleukin-6 in the control of acute and chronic *Giardia lamblia* infections in mice. Infect Immun (2003) 71:1566–8. 10.1128/iai.71.3.1566-1568.2003 12595478 PMC148826

[36] Bienz M Dai WJ Welle M Gottstein B Muller N. Interleukin-6-deficient mice are highly susceptible to *Giardia lamblia* infection but exhibit normal intestinal immunoglobulin A responses against the parasite. Infect Immun (2003) 71:1569–73. 10.1128/iai.71.3.1569-1573.2003 12595479 PMC148820

[37] Zhou P Li E Shea-Donohue T Singer SM Tumour necrosis factor α contributes to protection against *Giardia lamblia* infection in mice. Parasite Immunol (2007) 29:367–74. 10.1111/j.1365-3024.2007.00953.x 17576366 PMC2443547

[38] Li E Zhou P Petrin Z Singer SM. Mast cell-dependent control of *Giardia lamblia* infections in mice. Infect Immun (2004) 72:6642–9. 10.1128/IAI.72.11.6642-6649.2004 15501797 PMC523017

[39] Matowicka-Karna J Dymicka-Piekarska V Kemona H. IFN-gamma, IL-5, IL-6 and IgE in patients infected with *Giardia intestinalis*. Folia Histochem Cytobiol (2009) 47:93–7. 10.2478/v10042-009-0013-3 19419945

[40] Owen RL Allen CL Stevens DP. Phagocytosis of *Giardia muris* by macrophages in Peyer’s patch epithelium in mice. Infect Immun (1981) 33:591–601. 7275318 10.1128/iai.33.2.591-601.1981 PMC350740

[41] Hill DR Pohl R. Ingestion of *Giardia lamblia* trophozoites by murine Peyer’s patch macrophages. Infect Immun (1990) 58:3202–7. 2401561 10.1128/iai.58.10.3202-3207.1990 PMC313640

[42] Kamda JDT A Role for Dendritic Cells in Giardia lamblia Infection. Georgetown University (2007).

[43] Banik S Renner Viveros P Seeber F Klotz C Ignatius R Aebischer T. *Giardia duodenalis* arginine deiminase modulates the phenotype and cytokine secretion of human dendritic cells by depletion of arginine and formation of ammonia. Infect Immun (2013) 81:2309–17. 10.1128/IAI.00004-13 23589577 PMC3697621

[44] Kamda JD Nash TE Singer SM. *Giardia duodenalis*: dendritic cell defects in IL-6 deficient mice contribute to susceptibility to intestinal infection. Exp Parasitol (2012) 130:288–91. 10.1016/j.exppara.2012.01.003 22248985 PMC3289762

[45] Dong C Davis RJ Flavell RA MAP kinases in the immune response. Annu Rev Immunol (2002) 20:55–72. 10.1146/annurev.immunol.20.091301.131133 11861597

[46] Gowda DC. TLR-mediated cell signaling by malaria GPIs. Trends Parasitol (2007) 23:596–604. 10.1016/j.pt.2007.09.003 17980663

[47] Lee HY Hyung S Lee NY Yong TS Han SH Park SJ Excretory-secretory products of *Giardia lamblia* induce interleukin-8 production in human colonic cells via activation of p38, ERK1/2, NF-kappaB and AP-1. Parasite Immunol (2012) 34:183–98. 10.1111/j.1365-3024.2012.01354.x 22224945

[48] Manukyan MC Weil BR Wang Y Abarbanell AM Herrmann JL Poynter JA The phosphoinositide-3 kinase survival signaling mechanism in sepsis. Shock (2010) 34:442–9. 10.1097/SHK.0b013e3181e14ea9 20386497

[49] Guha M Mackman N. The phosphatidylinositol 3-kinase-Akt pathway limits lipopolysaccharide activation of signaling pathways and expression of inflammatory mediators in human monocytic cells. J Biol Chem (2002) 277:32124–32. 10.1074/jbc.M203298200 12052830

[50] Fukao T Tanabe M Terauchi Y Ota T Matsuda S Asano T PI3K-mediated negative feedback regulation of IL-12 production in DCs. Nat Immunol (2002) 3:875–81. 10.1038/ni825 12154357

[51] Martin M Schifferle RE Cuesta N Vogel SN Katz J Michalek SM. Role of the phosphatidylinositol 3 kinase-Akt pathway in the regulation of IL-10 and IL-12 by *Porphyromonas gingivalis* lipopolysaccharide. J Immunol (2003) 171:717. 10.4049/jimmunol.171.2.717 12847238

[52] Scott KG Yu LC Buret AG. Role of CD8+ and CD4+ T lymphocytes in jejunal mucosal injury during murine giardiasis. Infect Immun (2004) 72:3536–42. 10.1128/IAI.72.6.3536-3542.2004 15155662 PMC415705

[53] Liu S Jia H Hou S Zhang G Xin T Li H Recombinant TB10.4 of *Mycobacterium bovis* induces cytokine production in RAW264.7 macrophages through activation of the MAPK and NF-κB pathways via TLR2. Mol Immunol (2014) 62:227–34. 10.1016/j.molimm.2014.06.026 25019567

[54] Lawrence T Gilroy DW Colvillenash PR Willoughby DA. Possible new role for NF-kappaB in the resolution of inflammation. Nat Med (2001) 7:1291–7. 10.1038/86397 11726968

[55] Hayden MS Ghosh S. Shared principles in NF-kappaB signaling. Cell (2008) 132:344–62. 10.1016/j.cell.2008.01.020 18267068

[56] Bartelt LA Roche J Kolling G Bolick D Noronha F Naylor C Persistent *G. lamblia* impairs growth in a murine malnutrition model. J Clin Invest (2013) 123:2672–84. 10.1172/JCI67294 23728173 PMC3668820

